# Use of Physician Concerns and Patient Complaints as Quality Assurance Markers in Emergency Medicine

**DOI:** 10.5811/westjem.2016.9.30578

**Published:** 2016-10-07

**Authors:** Kiersten L. Gurley, Richard E. Wolfe, Jonathan L. Burstein, Jonathan A. Edlow, Jason F. Hill, Shamai A. Grossman

**Affiliations:** Harvard Medical School, Beth Israel Deaconess Medical Center, Department of Emergency Medicine, Boston, Massachusetts

## Abstract

**Introduction:**

The value of using patient- and physician-identified quality assurance (QA) issues in emergency medicine remains poorly characterized as a marker for emergency department (ED) QA. The objective of this study was to determine whether evaluation of patient and physician concerns is useful for identifying medical errors resulting in either an adverse event or a near-miss event.

**Methods:**

We conducted a retrospective, observational cohort study of consecutive patients presenting between January 2008 and December 2014 to an urban, tertiary care academic medical center ED with an electronic error reporting system that allows physicians to identify QA issues for review. In our system, both patient and physician concerns are reviewed by physician evaluators not involved with the patients’ care to determine if a QA issue exists. If a potential QA issue is present, it is referred to a 20-member QA committee of emergency physicians and nurses who make a final determination as to whether or not an error or adverse event occurred.

**Results:**

We identified 570 concerns within a database of 383,419 ED presentations, of which 33 were patient-generated and 537 were physician-generated. Out of the 570 reports, a preventable adverse event was detected in 3.0% of cases (95% CI = [1.52–4.28]). Further analysis revealed that 9.1% (95% CI = [2–24]) of patient complaints correlated to preventable errors leading to an adverse event. In contrast, 2.6% (95% CI = [2–4]) of QA concerns reported by a physician alone were found to be due to preventable medical errors leading to an adverse event (p=0.069). Near-miss events (errors without adverse outcome) trended towards more accurate reporting by physicians, with medical error found in 12.1% of reported cases (95% CI = [10–15]) versus 9.1% of those reported by patients (95% CI = [2–24] p=0.079). Adverse events in general that were not deemed to be due to preventable medical error were found in 12.1% of patient complaints (95% CI = [3–28]) and in 5.8% of physician QA concerns (95% CI = [4–8]).

**Conclusion:**

Screening and systemized evaluation of ED patient and physician complaints may be an underutilized QA tool. Patient complaints demonstrated a trend to identify medical errors that result in preventable adverse events, while physician QA concerns may be more likely to uncover a near miss.

## INTRODUCTION

Medical error is a correctable cause of morbidity and mortality. In 1991, the Harvard Medical Practice Study found that nearly 3.7% of admitted patients suffered complications from treatment, two-thirds of which were due to errors in care, and a significant portion of these were preventable.^1^, ^2^ This landmark study prompted intense national scrutiny of medical errors, which remain a significant burden.^3^, ^4^ Recent data indicate that the incidence of adverse events attributable to medical error among hospitalized patients may be increasing. Existing evidence supports a compelling argument for emergency departments (ED) to have systems in place to perform root cause analysis of potential errors, and to implement systemic corrections to improve care when such errors are found.^5^

Although it is clearly worthwhile to uncover medical error within the ED, an ideal marker for efficient error correction has yet to be uncovered. Twice each month, the ED quality assurance (QA) team screens all cases that meet certain empirically selected criteria, such as death within 24 hours, transfer from initial floor bed to ICU within 24 hours, physician self-reported concerns, nursing incident reports or cases that generate physician or patient complaints. These surrogates are often used as routine metrics in emergency medicine QA and although they are often perceived as the gold standard, they remain largely unvalidated expert opinion.^6^

A quantitative analysis evaluating the utilization of physician and patient complaints has not been studied. The presence of an integrated, readily accessible electronic error reporting system has facilitated the study of such measures in one urban tertiary care ED. The objective of this study was to determine whether systematic screening and evaluation of documented patient and physician QA concerns is a useful tool for identifying physician errors resulting in either an adverse or near-miss event.

## METHODS

### Study Design and Setting

We conducted a retrospective cohort study of consecutive patients presenting to an urban, tertiary care academic medical center ED with an annual volume of ~57,000 patients between January 2008 and December 2014. This ED maintains a QA database linking all patient and physician complaints to all patients.

To facilitate QA audits, a secure web-based platform was implemented in 2008 to automate a number of the reporting processes that were previously carried out by hand or through the use of photocopied patient documentation. The automated QA dashboard performs nightly sweeps of the computerized ED patient log to identify cases that meet predetermined criteria for QA review including deaths within 24 hours of ED arrival, return visits within 72 hours requiring hospitalization and floor admissions transferred to ICU within 24 hours, as well as cases involving high-risk procedures, such as endotracheal intubation or procedural sedation. There is a mechanism in place where physicians can flag cases for review on the QA dashboard. Alternatively, patients are able to report complaints through the hospital’s patient relations office. After automatic identification, or identification via a physician concern or patient complaint, the cases are assigned randomly to a physician reviewer from within the ED who was not involved in the care of the patient. To ensure that all reviewers receive similar numbers and a similar distribution of types of cases, cases are assigned with load balancing. A case detail page containing key demographic and operational data elements as well as relevant clinical data associated with the case is extracted from relevant hospital databases. The electronic scanned copy of all of the paper documentation associated with each case is captured from our billing process and stored in the electronic dashboard database.

The reviewers are notified automatically by email when a new case has been assigned to them. They are then able to log onto the QA dashboard and securely review the case documentation. Reviewers are also able to assess relevant records from the patients’ online medical records through embedded links in the case detail page.

After reviewing the case documentation, reviewers are then asked to respond to a series of seven standardized questions with answers formulated by a standardized Likert scale (see Figures 3 and 4 for examples), adding additional text comments as needed. If after case analysis, the reviewer has concerns about possible errors, adverse events or other quality issues, the case is referred for discussion by the full QA committee. At bimonthly meetings, the committee makes the final determination about whether error or adverse events occurred based on committee consensus. At the conclusion of each review and remediation process, all data elements are entered into the QA dashboard archive to be used for reference, quality improvement and research.

### Definition of Terms

The hospital’s institution-wide definition of medical error is the failure of a planned action to be completed as intended or the use of a wrong plan to achieve an aim. An adverse event is defined as unintended physical injury and/or physiologic insult resulting from or contributed to by medical care (including the absence of indicated medical treatment), that requires or prolongs hospitalization, and/or results in permanent disability or death that cannot be solely and definitively due to the progression of the patient’s underlying condition. Adverse events caused by medical error are termed preventable adverse events. Near-miss events are medical errors that do not result in an adverse event.^7^

### Selection of Participants

**Figure 1:**
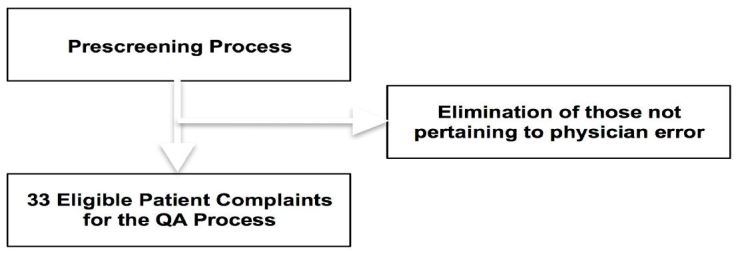
Patient complaints are prescreened to identify possible medical errors or adverse events. *QA,* quality assurance.

**Figure 2:**
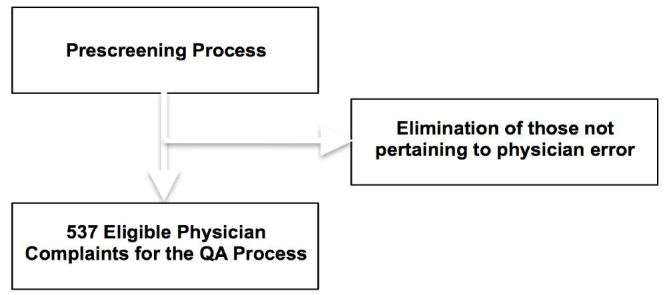
Physician reports are prescreened to identify possible medical errors or adverse events. *QA,* quality assurance.

We included all patients presenting to the ED during the specified period. Patient complaints refer to post-visit telephone or written complaints brought before the department chairs. Patient complaints are initially prescreened by an experienced evaluator and those not pertaining to possible medical error, such as complaints related to billing, creature comfort, communication, nursing related complaints and waiting times were eliminated. If a potential QA issue is present, the case is referred to the QA committee as illustrated in Figure 1. The ED has an electronic error reporting system that allows attending physicians or QA directors from all departments to register a concern or identify a potential QA issue via an easily accessible online form for subsequent review as illustrated in Figure 2. For lack of a better term, these “physician complaints” are then entered into an automated electronic QA database that interfaces with a commercially available HIS system that randomly assigns the patient and physician concerns to members of the QA panel to be reviewed by physician evaluators not involved with the patient’s care as described above.^8^

### Outcome Measures

The ED dashboard system lends itself to a one-click “flag” system for QA referral so any practitioner can easily identify a case for QA review. Once identified, the ED chair or QA director will review the complaint and, assuming it is related to quality improvement (QI), it will be entered into a QA database and undergo systematic review by a 20-member QA committee. The committee is comprised of emergency physicians and nurses who then give a final determination as to whether or not an error occurred. Ultimately, we compared the incidence of error and adverse events from flagged cases that initially linked both patient and physician complaints to more traditional markers, including 72 hour returns and floor to ICU transfer from our institution.

### Data Collection and Processing

**Figure 3:**
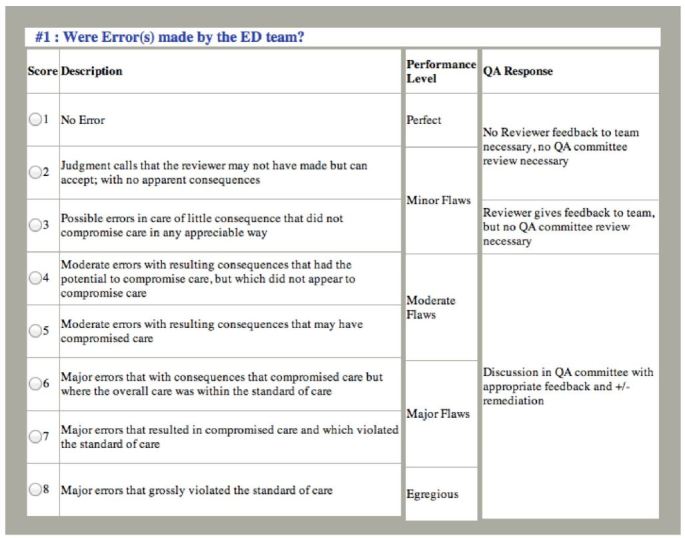
Standardized tool used by reviewers to determine presence of medical error in quality assurance cases. *QA,* quality assurance.

**Figure 4:**
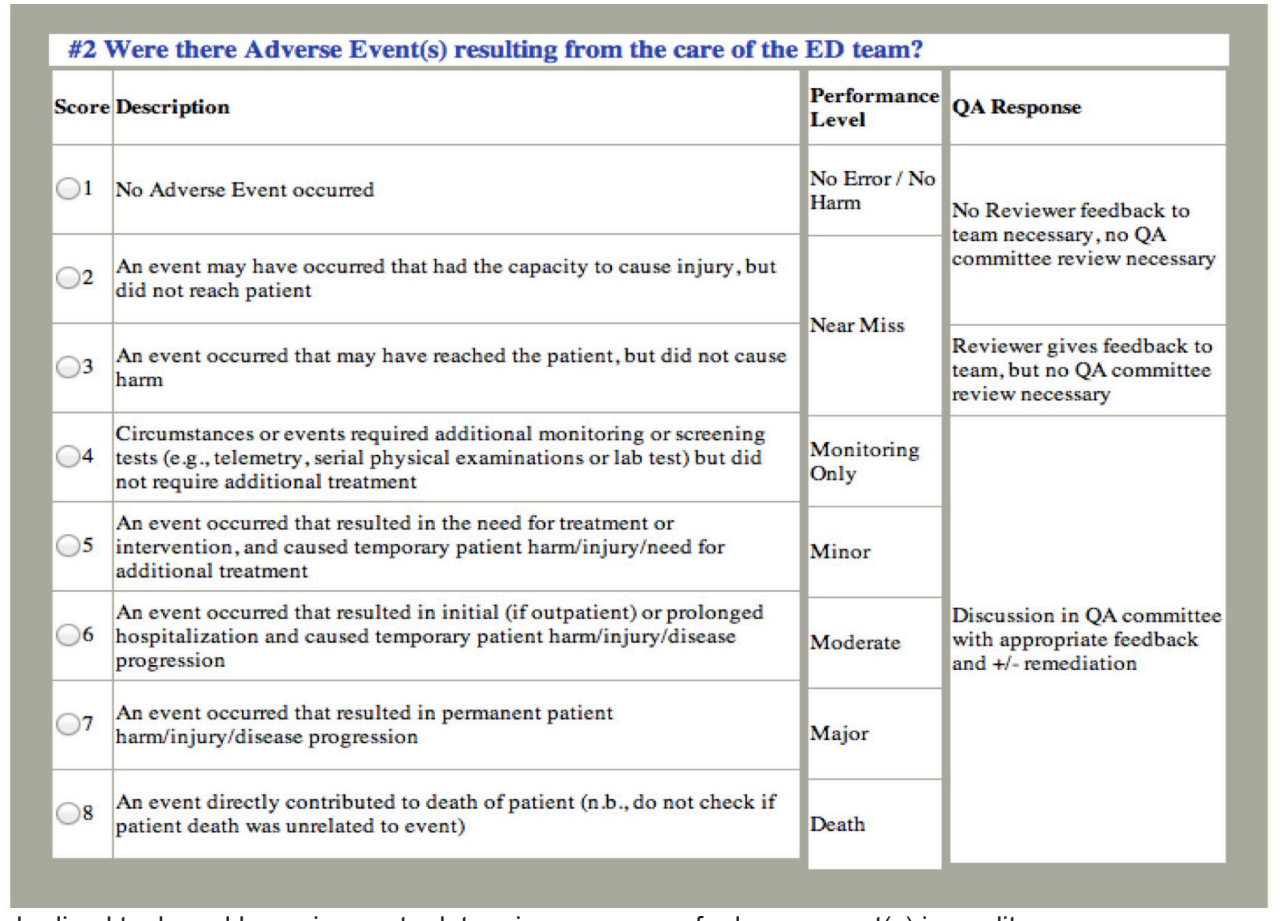
Standardized tool used by reviewers to determine presence of adverse event(s) in quality assurance cases. *QA,* quality assurance.

Physician evaluators are emergency medicine attending physicians who are trained via an online module and undergo an initial double review to evaluate cases for the occurrence of an error, adverse event, or a near-miss event. Cases are reviewed independently by reviewers who are not involved in the care of the given patient. Reviewers use a structured tool to determine the presence of error and adverse events using an eight-point Likert scale. A level of four, (corresponding to moderate error with resulting consequences that had the potential to compromise care, but which did not compromise care) or greater warrants full committee review. See Figures 3 and 4 for representatives of the Likert scale and a description of the first two of eight questions evaluated. The evaluating physician presents the case to the QA committee at their monthly meeting and the committee makes a final determination as to whether or not an error and/or adverse event occurred for each case.^9^

**Figure 5:**
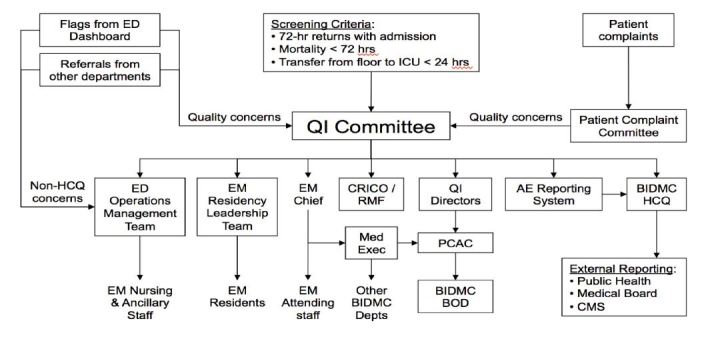
Structural schematic of how quality assurance issues are referred to different departments within the hospital. *QI,* quality improvement; *CRICO,* malpractice insurance program; *RMF*, risk management facility; *BOD;* board of directors; *PCAC,* Department Chiefs Quality Assurance Committee; *HCQ*, health care quality; *EM*, emergency medicine; *ED*, emergency department.

The ED’s QA committee is formally integrated into the hospital’s overall QA operations. Depending on outcomes of the review, the ED QI committee then refers its results for departmental corrective action and/or further action depending on the type of error. The findings may be forwarded for internal review, chief review, chairmen of departments review, hospital wide board of director review, or finally to the medical board or risk management services. See Figure 5 for a detailed schematic of the overall QI system and its integration into the hospital wide infrastructure.

### Statistical Analysis

Data were extracted from the QA database and entered into a Microsoft Excel 2003 (Redmond, WA) database program. We reported The rate of preventable adverse events, near-miss events and overall adverse events for patient and physician concerns with corresponding 95% confidence intervals using a Fishers-exact test. This method uses mathematical simulation to determine the likelihood of our findings occurring by chance. Results are reported as percentages.

## RESULTS

We identified 570 complaints within a database of 383,419 ED presentations, of which 33 were patient-generated and 537 were physician-generated. In the combined total complaints physician errors that led to a preventable adverse event were detected in 3.0% (95% CI = [1.52–4.28]). Further analysis revealed that 9.1% of patient concerns correlated to preventable errors leading to an adverse event (95% CI = [2–24]). In contrast, 2.6% of complaints made by a physician alone were found to be preventable medical errors leading to an adverse event (95% CI = [2–4] p=0.069). Near-miss events (errors without adverse outcome) showed a trend to be more accurately reported by physicians, with medical error found in 12.1% of physician-reported cases (95% CI = [10–15]) and in 9.1% of those reported by patients (95% CI = [2–24] p=0.79). Adverse events in general that were not deemed to be due to preventable medical error were found in 12.1% of patient complaints (95% CI = [3–28]) and in 5.8% of physician complaints (95% CI = [4–8]) (Table 1). When compared to our departmental near-miss and adverse event rates for 72 hour returns, floor to ICU transfers and procedural sedations; the use of patient and physician complaints as markers is comparable to the more standard metrics listed below. For 72 hour returns our near-miss rate is 10.2%, with an overall adverse event rate of 8.6%. Our floor to ICU transfer rate is 10.2% with a corresponding overall adverse event rate of 8.5%. We do not have data on preventable adverse event rates for these other markers at this time (Table 2).

## DISCUSSION

There is an ongoing need to improve and find new and more informative ED-based QA markers for clinical error, especially preventable error resulting in harm. In our study, we examined two markers, physician concerns and patient complaints to gauge their utility in routine QA review of ED patient care. We found the overall error rate was within expected ranges, 12.1% in those cases referred by patients and in 5.8% of those cases referred by physicians. When compared to more standard metrics such as floor to ICU transfer or 72 hour returns, physician and patient complaints appear to perform well in our initial analyses; however, we were not able to identify statistically significant differences between physician reports and patient complaints in identifying preventable adverse events or near-miss events. Physician reports had a trend towards a lower incidence of identifying adverse events associated with error when compared to patient complaints.

Medical error has received increased national attention over the last 20 years. Anderson et al. showed an overall incidence of error at 0.13% in ED care.^6^ Overall, there is a dearth of high-quality evidence describing the incidence of error and adverse events in the ED.^10^ The Anderson study, reviewing only physician complaints about ED patient care, found that 22.6% of the errors identified were identified by complaints and 19.9% of adverse events were identified by complaints, although the proportion that were preventable was not reported.^6^

Prior investigations suggest that systematic evaluation of physician complaints have been shown to have a high yield for detecting error.^6^ Patients complaints, however, have yet to be formally evaluated. Peer review may be a logical approach for discerning error and adverse events among physicians in medicine given the requisite specialized knowledge base and expertise. Therefore, one could assume that physician complaints would be a superior primary source for uncovering adverse events and error in medicine, yet there is limited literature looking at physician complaints as a marker for QA. Recent investigations suggest that physician complaints have a high yield for detecting error. 6 Paradoxically, the ability of our patients to recognize physician error without the requisite training in medicine was studied here and found to be a useful QA metric. It is possible that subjective involvement of the patient, although open to bias, may be more useful than objective evaluation in recognizing error.

Finally, we looked at preventable adverse events, which is a patient-centered outcome. Patient complaints appeared to provide useful information in identifying preventable adverse events. The ultimate goal of such detection is to implement system-based changes to decrease future error. Our findings show promise for tracking both physician and patient complaints as high-yield markers of QA-relevant events.

## LIMITATIONS

By using an initial single physician pre-screener for each patient complaint, relevant cases may have been missed since this is an inherently subjective process. To mitigate this potential limitation, we reviewed a random sample of patient complaint cases that were not brought for QA committee review and found these cases involved complaints that do not pertain to physicians (such as lack of warm blankets) or involve ancillary staff (which is another area deserving further scrutiny). We also used a single institution for a test site, which may limit the generalizability of the conclusions of this study. Lastly, the sample size of this study was small especially for the patient complaint side, perhaps implying hesitancy on the part of the patient to report possible error. Such a small sample size may lead to statistical errors. The study is likely underpowered and may contribute to a type 1 error, where a true difference may not be identified. Finally, there was lack of long-term follow up in these patients, which may have been another opportunity to identify further errors or adverse outcomes. Further research with larger sample sizes should be performed when possible.

## CONCLUSION

Screening and systematic evaluation of ED patient complaints and physician concerns may be an underused and efficient QA tool. Patient complaints may accurately identify medical errors that result in preventable adverse events. Physician concerns may be more likely to uncover a near miss that did not lead to an adverse event. Both patient and physician complaints may be useful QA metrics for identifying error in ED care when compared to routine metrics such as 72 hour returns and floor to ICU transfer.

## Figures and Tables

**Table 1:** Comparison of percentage of physician reports and patient complaints reviewed by the QA committee that identified a preventable adverse event or near-miss event.

	Patient complaints; n=33 error rates	Physician concerns; n=537 error rates	P-value
Preventable adverse event	3(9.1%)	14(2.6%)	0.069
Near miss event	3(9.1%)	65(12.1%)	0.79
Overall adverse event	4(12.1%)	31(5.8%)	0.136

*QA,* quality assurance.

**Table 2:** Comparison between standard metrics versus physician reports and patient complaints of identifying adverse events and near misses.

	Near miss rate	Adverse event rate
72-hour returns	10.2%	8.6%
Floor to ICU transfer	10.2%	8.5%
Procedural sedation	1.9%	0%
Physician complaints	12.1%	5.8%
Patient complaints	9.1%	12.1%

*ICU,* intensive care unit
